# Cannabidiol Oil-Associated Microscopic Colitis

**DOI:** 10.7759/cureus.10528

**Published:** 2020-09-18

**Authors:** Poornima Oruganti, Stephanie Betcher, Zuie Wakade, Xianzhong Ding, Ayokunle T Abegunde

**Affiliations:** 1 Internal Medicine, Loyola University Medical Center, Maywood, USA; 2 Gastroenterology, Loyola University Medical Center, Maywood, USA; 3 Pathology, Loyola University Medical Center, Maywood, USA

**Keywords:** cannabis, diarrhea, microscopic colitis

## Abstract

Microscopic colitis (MC) is a chronic inflammation condition of the colon characterized by watery diarrhea and normal appearing mucosa. A 75-year-old female presented with one-year history of chronic diarrhea while taking cannabidiol (CBD) for pain. Colonoscopy with random colon biopsies revealed collagenous colitis. She started budesonide and stopped CBD. At six-week follow-up, her diarrhea improved, and the budesonide dose was decreased. She restarted CBD oil twice but had diarrhea both times. Her diarrhea resolved after taking budesonide and stopping CBD. We report a case of CBD-associated MC to make clinicians aware of this potential adverse effect in patients who chronically use CBD.

## Introduction

Microscopic colitis (MC) is a chronic inflammation condition of the colon characterized by watery, non-bloody diarrhea and generally normal appearing colonic mucosa on colonoscopy. Colon biopsy is required to confirm the diagnosis and differentiate between the two subtypes: lymphocytic and collagenous colitis (CC) [1]. Many drugs have been associated with MC. However, the pathophysiology is incompletely understood. Drugs that have been implicated in MC include non-steroidal anti-inflammatory drugs (NSAIDs), aspirin, proton-pump inhibitors (PPIs), selective serotonin reuptake inhibitors (SSRIs), clozapine, and acarbose [2]. Studies examining the etiology of MC are limited and mostly consist of case reports and observational studies. Cannabidiol (CBD) is a concentrated oily residue of the plant Cannabis sativa. Recent changes in the legality of CBD have led to an increased acceptance of its use by the medical community. The Controlled Substances Act (CSA) of 1970 made the growth of hemp and marijuana illegal in the United States, but in 2014, the Agricultural Act allowed for industrial growth of hemp [3]. CBD and hemp are different from marijuana because they have low tetrahydrocannabinol (THC) levels [3]. This provides patients with the medical benefits of Cannabis without the intoxicating effects of marijuana [3]. CBD has found some popularity in the search for an alternative to opioids for the treatment of pain [4]. CBD is not regulated by the U.S. Food and Drug Administration (FDA), and there are no determinations on appropriate dosage, safety, efficacy, or interactions with other drugs or food [3]. There are currently no reports in the medical literature that CBD is a risk factor for the development of MC. We report a case of CBD-associated MC to make clinicians aware of this potential adverse effect in patients who chronically use CBD. The patient agreed to the use and publication of her disease process and case with her personal health information deleted.

## Case presentation

**Figure 1:**
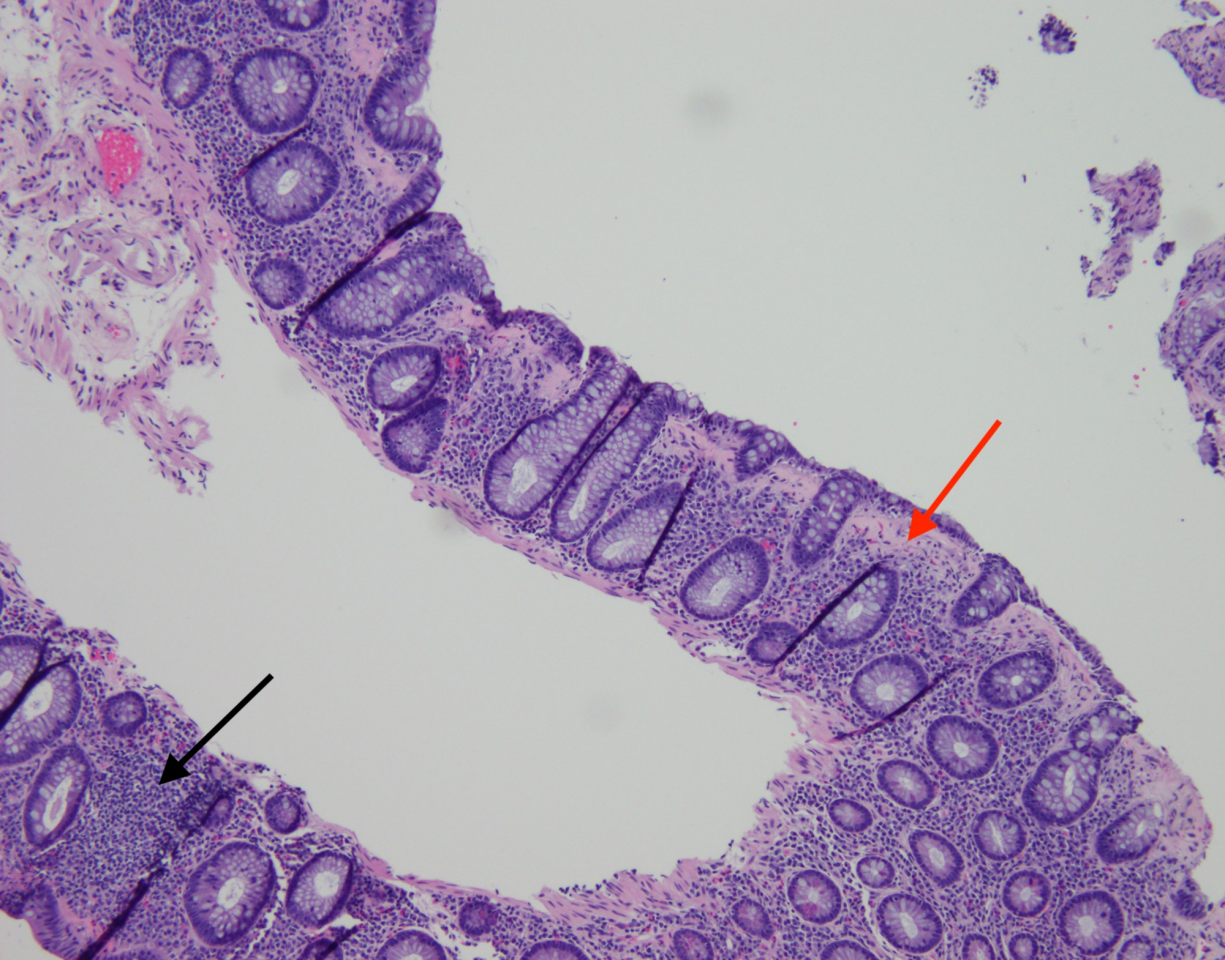
H&E stain showing increased intraepithelial lymphocytes (black arrow) and subepithelial collagen band > 10 microns (red arrow) consistent with collagenous colitis.

**Figure 2:**
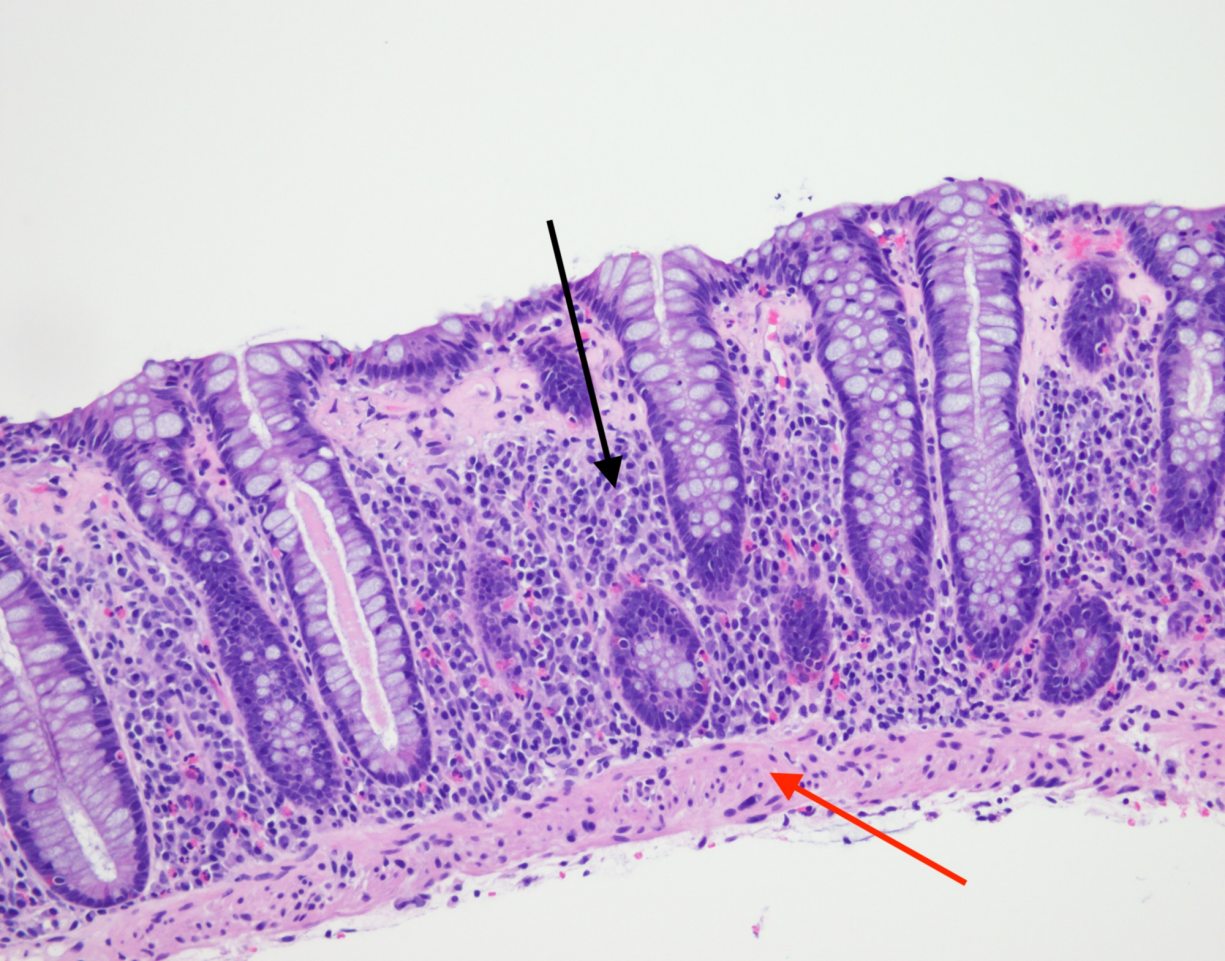
H&E stain showing increased intraepithelial lymphocytes (black arrow) and subepithelial collagen band >10 microns (red arrow) consistent with collagenous colitis.

A 75-year-old Caucasian female with a history of anemia, hypothyroidism, and migraines presented to the gastroenterology clinic with a one-year history unexplained diarrhea. The patient started taking CBD oil for lumbosacral pain about one year prior to presentation. She endorsed watery, non-bloody, nocturnal diarrhea, left lower quadrant abdominal pain, and unintentional weight loss of eight pounds. The patient also endorsed urgency, tenesmus, fecal incontinence, and a small amount of blood when wiping after bowel movements (BMs). She had already tried loperamide and bismuth subsalicylate. She also tried the BRAT (bananas, rice, applesauce, and toast) diet and probiotic yogurt, but neither diet nor over-the-counter medications were effective in reducing her diarrhea. Prior to onset of diarrhea, the patient had constipation, which required her to take metamucil to have one BM daily. However, she began to have two loose BMs daily, and then her stool frequency increased to five times daily and three to four times at night. Colonoscopy performed for polyp surveillance six months prior to presentation and symptom onset revealed decreased anal sphincter tone and multiple colon polyps; grossly, mucosa appeared normal and random biopsies were not taken. Repeat colonoscopy with random biopsies six months later (at symptom onset) revealed CC (Figures 1, 2). She was still taking CBD oil at the time of her second colonoscopy demonstrating MC. She was not taking NSAIDs or any other agent associated with MC at the time of her second colonoscopy. Stool PCR was negative for enteric pathogens. Budesonide therapy was started, and she was advised her to continue a high fiber diet and to avoid artificial sweeteners and sugar alcohols. She was advised to avoid NSAIDs and discontinue CBD oil. During follow-up six weeks later, her diarrhea had improved. Her gastroenterologist reduced the dose of budesonide. After her six-week follow-up, the patient started taking CBD oil again at home and had a recurrence of diarrhea. She stopped CBD oil, and her diarrhea resolved. One week later, she restarted CBD oil and experienced diarrhea again. She then stopped CBD oil completely and completed a tapering course of budesonide. Eventually, diarrhea turned to constipation. She was advised to continue fiber supplements and high fiber diet. About three months after the initial presentation, she was able to stop budesonide. There was no recurrence of diarrhea after she stopped taking CBD oil and completed budesonide therapy.

## Discussion

The cause-effect relationship between drugs and MC is difficult to define. The patient in this case used a CBD soft gel made with hemp oil, extra virgin olive oil, vegetarian soft gel (vegetable cellulose, water), and silica. However, there are multiple different formulations of CBD oil and capsules, as well as other products including honey, vape pens, teas, gelatin snacks, baked goods, and beverages. Experimental studies on murine colitis suggest that physiologically relevant concentrations of exogenous CBD can reduce gut inflammation by stimulating CB1/CB2 cannabinoid receptors and endogenous cannabinoids N-arachidonoyl-ethanolamine (anandamide) and 2-arachidonoylglycerol (2-AG) [5,6]. However, data are limited on dose-related adverse effects of CBD. As the popularity of CBD products increases, more research is necessary regarding their safety and efficacy in humans. The World Health Organization (WHO) has proposed a method that establishes causality based on temporal sequence, prior information on the drug, dose-response relationship, pattern of response to the drug, the re-challenge, exclusion of other alternative etiologic candidates, and exposure to concomitant drugs. The events are then classified as “certain, probable, possible, unlikely, and not assessable” (Table 1) [2,7]. This case demonstrates a temporal relationship between exposure to CBD oil and onset of diarrhea, resolution of diarrhea after withdrawal of CBD oil, and reoccurrence of diarrhea with two re-challenge attempts with CBD oil while the patient was taking budesonide. Therefore, we can surmise that her CC resulted from chronic ingestion of CBD oil. The mechanism by which CBD oil induced MC in this case is unclear; however, we hypothesize that elevation of tissue levels of endocannabinoids may cause colonic inflammation by stimulating the vanilloid receptor subtype 1 (VR1) to release substance P [8]. Based on the WHO method, we believe that this case establishes a causal link between CBD and MC that can be classified as "probable or likely". To our knowledge there are currently no other case reports that describe such a relationship between CBD and MC. We recommend that physicians educate themselves on CBD containing products, and encourage open communication with patients regarding dietary supplements and their potential clinical adverse effects. 

**Table 1:** WHO Drug Causality Assessment System (Adapted From WHO-UMC*) *WHO-UMC, World Health Organization-Uppsala Monitoring Centre

Causality Term	Assessment Criteria
Certain	Plausible time relationship between event and drug intake and response to withdrawal
	Event definitive objectively, meaning pharmacologically or phenomenologically
	Event cannot be explained by disease or other drugs
	Re-challenge satisfactory, if necessary
Probable/Likely	Reasonable time relationship between event and drug intake and response to withdrawal
	Unlikely to be explained by disease or other drugs
	Re-challenge not required
Possible	Reasonable time relationship between event and drug intake and response to withdrawal
	Could also be explained by disease or other drugs
Unlikely	Improbable, but not impossible, time relationship between event and drug intake
	Disease or other drugs provide plausible explanation
Conditional/Unclassified	Event occurred, but more information is required
Unclassifiable	Insufficient data

## Conclusions

Multiple drugs have been implicated in the development of MC. CBD oil is a relatively new dietary supplement that is gaining popularity as an alternative to opioids for the treatment of pain. To our knowledge, there are currently no other case reports that describe such a relationship between CBD and MC. Physicians should suspect CBD-associated MC in patients taking CBD who develop diarrhea for which no other causes can be identified. We recommend that physicians educate themselves on CBD containing products and encourage open communication with patients regarding dietary supplements and their potential clinical adverse effects.
